# Characteristics of False-Positive Alarms in the BacT/Alert 3D System

**DOI:** 10.1128/spectrum.00055-22

**Published:** 2022-04-25

**Authors:** Misato Amano, Mami Matsumoto, Shigeru Sano, Mayumi Oyama, Hideto Nagumo, Naoko Watanabe-Okochi, Nelson H. Tsuno, Kazunori Nakajima, Kazuo Muroi

**Affiliations:** a Kanto-Koshinetsu Block Blood Center, Japanese Red Cross Society, Tokyo, Japan; b Central Blood Institute, Japanese Red Cross Society, Tokyo, Japan; University Paris-Saclay, AP-HP Hôpital Antoine Béclère, Service de Microbiologie, Institute for Integrative Biology of the Cell (I2BC), CEA, CNRS

**Keywords:** BacT/Alert, blood culture, false positive

## Abstract

The BacT/Alert system has been used for detecting the presence of bacteria in various clinical settings as well as in blood services, but it is associated with a relatively high incidence of false-positive results. We analyzed the results of our quality control sterility testing of blood products by BacT/Alert 3D to understand the mechanism of false-positive results. Anaerobic and aerobic bottles were inoculated with 10 mL of samples and cultured in BacT/Alert 3D for 10 days. Positive-reaction cases were classified as true positive if any bacterium was identified or false positive if the identification test had a negative result. The detection algorithm and the bottle graph pattern of the positive reaction cases were investigated. Among the 43,374 samples, 25 true positives (0.06%) and 29 false positives (0.07%) were observed. Although the detection algorithm of all true positives and 25 of 29 false positives was accelerating production of CO_2_, a steep rise in the bottle graph was observed only in the true positives, and it was not observed in either of the false positives. Four of 29 false positives were dependent on high baseline scatter reflections. Furthermore, evaluating the bottle graph pattern of Streptococcus pneumoniae, a bacterium known to autolyze, we confirmed that no viable bacterium was detected even if a steep rise was observed. In conclusion, the bottle graph pattern of positive reactions allows the differentiation between true positives and false positives. In case of a steep rise without bacterium detection, the bacterium might have autolyzed. Moreover, positive reactions with high baseline scatter reflections, despite immediate loading of bottles after sampling, are potentially false positive.

**IMPORTANCE** In clinical settings, false-positive results are treated as positive until bacterial identification. It may result in the discarding of blood products in blood centers or affect clinical decisions in hospitals or testing facilities. Moreover, the management of these samples is usually time- and labor-consuming. The results of our study may help clinicians and laboratory staff in making a more precise evaluation of positive reactions in BacT/Alert.

## INTRODUCTION

Bacterial contamination of blood products persists as a threat for recipients, especially immunosuppressed oncohematological patients. Recently, expanded screening protocols for platelet concentrates (PCs) have been implemented in many countries, including the United Kingdom and Canada (1, 2), with good results in terms of prevention of bacterial infection. With this technology implemented, the shelf-life of PC products could be extended to up to 7 days in the United Kingdom and Canada (1, 2). BacT/Alert 3D (bioMérieux, Marcy l'Étoile, France), which is based on the detection of CO_2_ emitted from bacteria in culture bottles, is the main method applied for bacterial screening in most countries.

In Japan, a different strategy has been implemented to secure the safety of PC products (3, 4). Instead of screening all PCs for the presence of bacteria, PC products have been supplied with a very short shelf-life of 3 days, compared to 5 days or longer in European countries, the United States, and Canada (1, 2, 5, 6). After the implementation of the diversion method at the time of PC collection, the incidence of contaminated PCs decreased by 71% (from 0.17% to 0.05%), as confirmed by testing more than 20,000 expired PCs (3). Also, red cell concentrates (RCCs) are supplied with a shelf-life of 20 days (7) compared to shelf-lives as long as 42 days in other countries (8–11). It is based on the following concept: the shorter the shelf-life of the product, the lower the risk of bacterial growth in the product during the storage. In addition, we conduct quality control sterility testing of our blood products at least once a month by sampling them at a constant rate.

However, even with this strategy, in 2017, we experienced 3 cases of sepsis after PC transfusion out of 834,051 PC products (909,271.5 units; 1 unit is equal to ≥2.0 × 10^11^ platelets [PLTs]/bag.) supplied, with a fatal consequence in 1 (12), and 4 cases out of 815,823 PC products (888,283.8 units) supplied in 2018 (7). Taking this result, discussions were started at the Japanese Red Cross Society (JRCS) on the implementation of bacterial screening of all PC products based on the United Kingdom and Canadian experiences.

Bacterial culture test by the BacT/Alert system is, however, known to be associated with a number of false-positive (13) and -negative (14) results. In Canada, bacterial screening has been reported to be associated with false-negative results in about 1/1,000 in the testing of outdated PCs, resulting in septic transfusion reactions in about 1/350,000 (2). Therefore, it is important to realize that culture systems reduce the risk of transfusion of contaminated PCs but cannot guarantee sterility (15–18). On the other hand, false-positive results lead to the discarding of PC products that would be potentially transfused. Thus, it is important to understand the frequency of these events, how they can be prevented, and how they can affect the supply of PC products.

In the present study, we analyzed and compared the bottle graph patterns of true-positive and false-positive samples identified by BacT/Alert 3D during the quality control sterility testing. In addition, we evaluated the bottle graph pattern of Streptococcus pneumoniae, a type of bacterium known to autolyze, with consequent negative bacterial identification after a positive test in the BacT/Alert system (19).

## RESULTS

### Analysis of the true-positive and false-positive cases in quality control sterility testing by BacT/Alert 3D.

Among the 43,374 cases tested during the 3-year period, 54 cases (0.12%) were positive. Twenty-five of 54 positives were positive in the bacterial identification test (0.06%), namely, true positive. On the other hand, 29 of 54 positives were negative for the bacterial identification test (0.07%), namely, false positive (Tables 1 and 2). The false positives accounted for 53.7% of the positive samples. The false-positive rate was 0.32% in the period from July 2015 to March 2016, but it decreased to 0.05% or less after April 2016 (Table 2).

**TABLE 1:** True- and false-positive rates by product

Product	No. of samples	No. of true-positive samples	% True positive	No. of false-positive samples	% False positive
RCCs	38,469	22	0.06	27	0.07
PCs	2,041	1	0.05	2	0.10
FFP	2,864	2	0.07	0	0.00
Total	43,374	25	0.06	29	0.07

**TABLE 2:** True- and false-positive rates by fiscal year

Period	No. of samples	No. of true-positive samples	% True positive	No. of false-positive samples	% False positive
2015.7–2016.3	4,421	6	0.14	14	0.32
2016.4–2017.3	12,024	4	0.03	4	0.03
2017.4–2018.3	19,826	11	0.06	10	0.05
2018.4–2018.7	7,103	4	0.06	1	0.01
Total	43,374	25	0.06	29	0.07

Of the 25 true positives, 22 cases were RCCs, 1 was PC, and 2 were fresh frozen plasma (FFP) (Table 3). Twenty-three cases of the 25 true positives were positive in anaerobic (BPN) bottles only, and 2 cases were positive in both BPN and aerobic (BPA) bottles. Cutibacterium acnes was identified in 20 of the 25 true-positive cases (80%). The time required for the positive reaction of C. acnes ranged from 2.71 to 7.71 days; the majority of which (15/20) gave positive reactions within 3.00 to 4.99 days. Except for the two anaerobic bacteria, namely, C. acnes and Anaerococcus prevotii, aerobic bacteria (*Bacillus* sp., Staphylococcus [coagulase-negative staphylococci], and Streptococcus pyogenes) were detected in 4 cases within 1 day of culture, ranging from 0.28 to 0.81 days.

**TABLE 3:** Time to positive reaction and type of bacteria identified among true-positive samples

Sample no.	Blood product^*a*^	Bottle type	Time to positive reaction (days)	Type of bacteria^*b*^
1	Ir-RCC-LR	BPN	2.91	Cutibacterium acnes
2	Ir-RCC-LR	BPN	3.29	Cutibacterium acnes
3	RCC-LR	BPN	3.04	Cutibacterium acnes
4	FFP-LR	BPN	0.61	*Bacillus* sp.
5	Ir-RCC-LR	BPN	3.06	Cutibacterium acnes
6	Ir-RCC-LR	BPN	2.84	Cutibacterium acnes
7	RCC-LR	BPN	0.73	Staphylococcus (CNS)
		BPA	0.81	Staphylococcus (CNS)
8	Ir-RCC-LR	BPN	4.46	Cutibacterium acnes
9	Ir-RCC-LR	BPN	3.94	Cutibacterium acnes
10	Ir-RCC-LR	BPN	4.84	Cutibacterium acnes
11	Ir-RCC-LR	BPN	3.86	Cutibacterium acnes
12	Ir-RCC-LR	BPN	3.12	Cutibacterium acnes
13	Ir-RCC-LR	BPN	4.41	Cutibacterium acnes
14	Ir-RCC-LR	BPN	3.79	Cutibacterium acnes
15	Ir-RCC-LR	BPN	3.34	Cutibacterium acnes
16	Ir-RCC-LR	BPN	2.71	Cutibacterium acnes
17	Ir-RCC-LR	BPN	3.72	Cutibacterium acnes
18	FFP-LR	BPN	7.71	Cutibacterium acnes
19	Ir-RCC-LR	BPN	5.17	Cutibacterium acnes
20	Ir-PC-LR	BPN	0.28	Streptococcus pyogenes
		BPA	0.31	Streptococcus pyogenes
21	Ir-RCC-LR	BPN	4.01	Cutibacterium acnes
22	Ir-RCC-LR	BPN	0.75	Staphylococcus (CNS)
23	Ir-RCC-LR	BPN	5.43	Anaerococcus prevotii
24	Ir-RCC-LR	BPN	4.03	Cutibacterium acnes
25	Ir-RCC-LR	BPN	4.80	Cutibacterium acnes

a (Ir-)RCC-LR, (irradiated) red cell concentrates-leukocyte reduction; (Ir-)PC-LR, (irradiated) platelet concentrates-leukocyte reduction; FFP-LR, fresh frozen plasma-leukocyte reduction.

b Bacterial identification was conducted at an external laboratory.

Among the 29 cases of false-positive reaction, 14 cases (48.3%) were positive in BPN and 15 (51.7%) in BPA (Table 4). The detection time ranged from 0.28 to 2.00 days in BPN and 0.08 to 2.69 days in BPA. There was no significant difference between BPN and BPA.

**TABLE 4:** Time to positive reaction among false-positive samples (screening-positive bottles)

Sample no.	Blood product^*a*^	Bottle type	Time to positive reaction (days)
1	Ir-RCC-LR	BPA	0.09
2	Ir-RCC-LR	BPA	1.34
3	Ir-RCC-LR	BPN	1.00
4	Ir-RCC-LR	BPN	0.98
5	Ir-RCC-LR	BPA	0.99
6	Ir-RCC-LR	BPA	2.05
7	Ir-RCC-LR	BPA	1.02
8	Ir-RCC-LR	BPN	2.00
9	Ir-RCC-LR	BPN	0.94
10	Ir-RCC-LR	BPN	0.99
11	Ir-RCC-LR	BPN	1.15
12	Ir-RCC-LR	BPN	1.15
13	Ir-RCC-LR	BPA	1.14
14	Ir-RCC-LR	BPN	0.98
15	Ir-RCC-LR	BPA	0.08
16	RCC-LR	BPN	1.19
17	Ir-RCC-LR	BPN	0.28
18	Ir-RCC-LR	BPN	1.19
19	Ir-PC-LR	BPA	0.96
20	Ir-RCC-LR	BPA	0.08
21	RCC-LR	BPA	0.08
22	Ir-RCC-LR	BPA	1.24
23	Ir-RCC-LR	BPN	1.23
24	Ir-RCC-LR	BPA	1.25
25	Ir-PC-LR	BPN	1.23
26	RCC-LR	BPA	1.23
27	Ir-RCC-LR	BPN	1.05
28	Ir-RCC-LR	BPA	2.69
29	Ir-RCC-LR	BPA	0.31

a (Ir-)RCC-LR, (irradiated) red cell concentrates-leukocyte reduction; (Ir-)PC-LR, (irradiated) platelet concentrates-leukocyte reduction; FFP-LR, fresh frozen plasma-leukocyte reduction.

**FIG 1:**
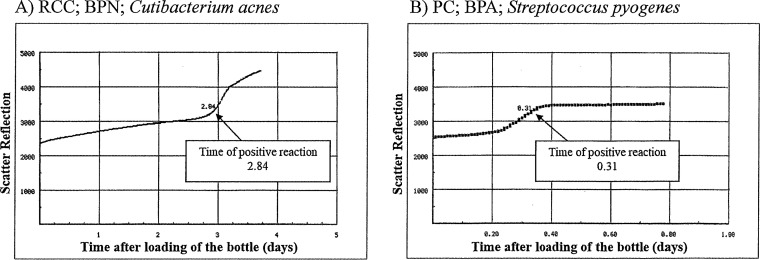
Examples of the bottle graph of true-positive cases. A steep rise is observed in the bottle graph. (A) RCCs, BPN bottles; Cutibacterium acnes was identified. (B) PCs, BPA bottles; Streptococcus pyogenes was identified.

**FIG 2:**
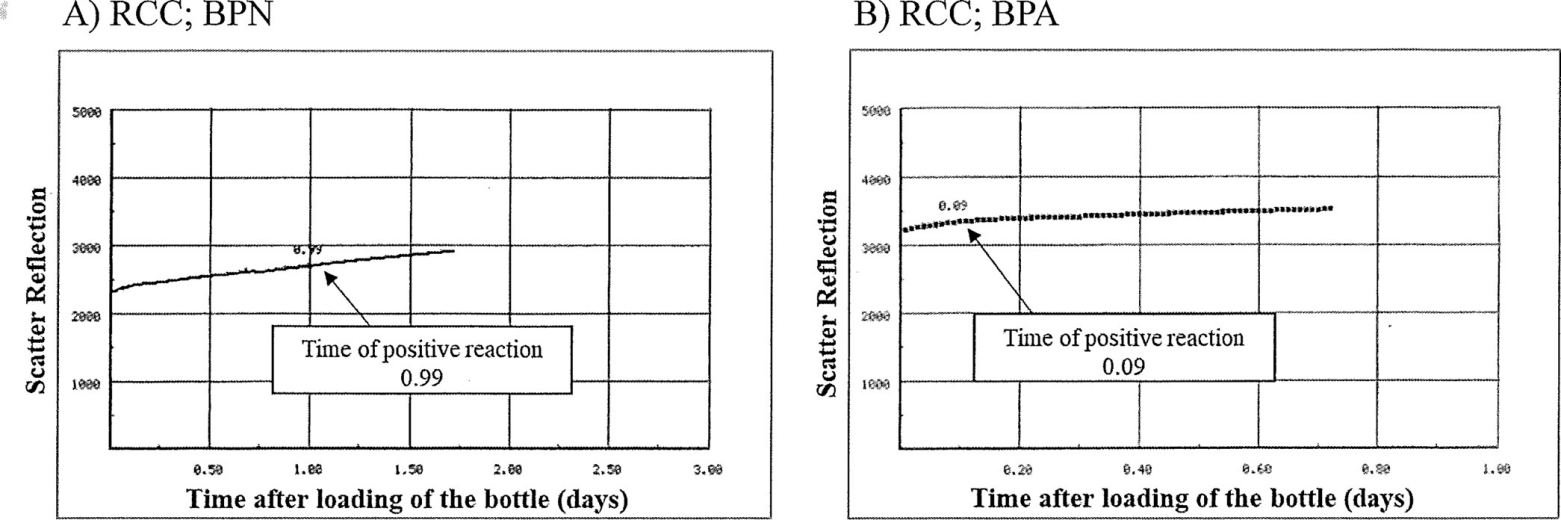
Examples of the bottle graph of false-positive cases. A steep rise in the bottle graph is not observed. (A) RCCs, BPN bottles; the detection algorithm is the accelerating production of CO_2_. (B) RCCs, BPA bottles; the detection algorithm is high initial CO_2_ content.

The detection algorithm of all 25 true-positive cases was accelerating production of CO_2_. A steep rise pattern was confirmed in the bottle graph of all samples (Fig. 1A and B), different from that of negative samples, which was flat. Although the detection algorithm of 25 out of 29 false-positive cases was also accelerating production of CO_2_, the steep rise was not observed (Fig. 2A). The detection algorism of the remaining 4 of the 29 false-positive cases was high initial CO_2_ content (Fig. 2B).

### Bottle graph pattern of the bottles inoculated Streptococcus pneumoniae.

**FIG 3:**
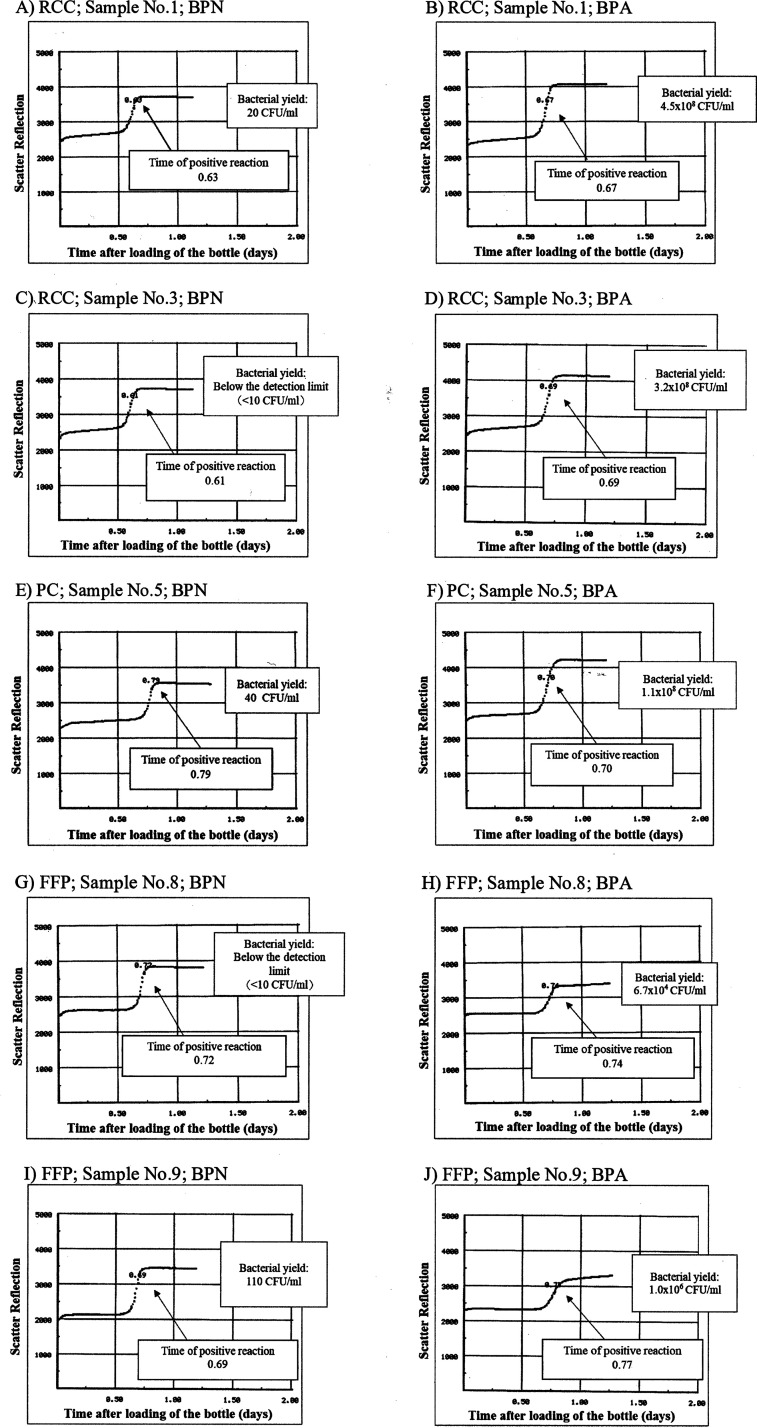
Bottle graphs of the sample inoculated with Streptococcus pneumoniae, showing the steep rise pattern of the CO_2_ elevation curves both in BPN and BPA. (A) RCCs, sample 1, BPN; (B) RCCs, sample 1, BPA; (C) RCCs, sample 3, BPN; (D) RCCs, sample 3, BPA; (E) PCs, sample 5, BPN; (F) PCs, sample 5, BPA; (G) FFP, sample 8, BPN; (H) FFP, sample 8, BPA; (I) FFP, sample 9, BPN; (J) FFP, sample 9, BPA. The bacterial yields 12 h after the positive signal was detected are also shown.

All bacteria-inoculated samples of RCCs, PCs, and FFP signaled positive both in BPN and BPA, and their detection algorithm was accelerating production of CO_2_ (Table 5). As with the true-positive cases of the quality control sterility testing, the CO_2_ elevation curve of the bottle graphs showed a steep rise pattern both in BPN and BPA (Fig. 3). The bacterial yield 12 h after the positive signal detection ranged from 3.6 × 10^4^ to 4.5 × 10^8^ CFU/mL in BPA bottles. On the other hand, the bacterial yield decreased in BPN bottles, with the highest level at 8.0 × 10^3^ CFU/mL, and the values were below the detection limit (<10 CFU/mL) in 1 of 3 RCC samples (Fig. 3C) and 2 of 3 FFP samples (Fig. 3G), despite the steep rise graph pattern. Furthermore, one sample of RCC (Fig. 3C), which showed a value below the detection limit, was also negative in the subculture, whereas the other remaining samples were all positive in the subculture bottles (Table 5). S. pneumoniae was identified by 16S rRNA gene sequencing in all the samples with a bacterial count below the detection limit.

**TABLE 5:** Results of the Streptococcus pneumoniae inoculation test

Sample type	Sample no.	Bottle type	Time to positive reaction (days)	Bacterial yield (CFU/mL)	Time to positive reaction in subculture (days)
RCCs	1	BPN	0.63	20	0.84
		BPA	0.67	4.5 × 10^8^	0.20
	2	BPN	0.69	8.0 × 10^3^	0.62
		BPA	0.73	1.1 × 10^8^	0.23
	3	BPN	0.61	Below the detection limit (<10)	Negative
		BPA	0.69	3.2 × 10^8^	0.17
PCs	4	BPN	0.78	70	0.69
		BPA	0.70	4.0 × 10^7^	0.27
	5	BPN	0.79	40	0.70
		BPA	0.70	1.1 × 10^8^	0.28
	6	BPN	0.80	3,030	0.56
		BPA	0.72	7.0 × 10^7^	0.33
FFP	7	BPN	0.68	Below the detection limit (<10)	0.79
		BPA	0.72	3.6 × 10^4^	0.31
	8	BPN	0.72	Below the detection limit (<10)	0.76
		BPA	0.74	6.7 × 10^4^	0.39
	9	BPN	0.69	110	0.66
		BPA	0.77	1.0 × 10^6^	0.30

## DISCUSSION

The detection algorithm of all true-positive samples in our study was accelerating production of CO_2_, with a steep rise pattern of the bottle graphs. Also, a similar pattern was observed in the samples inoculated with S. pneumoniae. On the other hand, although the detection algorithm of some false-positive samples was also accelerating production of CO_2_, the steep rise pattern of the bottle graphs was not observed in any of these samples. This result is compatible with the previous report by Hundhausen et al., although they used PC samples collected within 24 h, whereas we used longer-stored samples (20). Based on our standard operating procedures (SOPs), we inoculated the blood product only once, which is different from the procedures at other blood centers abroad, where the second sample of the blood product is inoculated into a new bottle to confirm the reproducibility of the positive reaction. To confirm our results, however, we inoculated the second samples from nine blood products suspected of false-positive results into new bottles and cultured them in BacT/Alert 3D in a separate experiment. We confirmed that all of the second samples had negative results. Therefore, in those samples signaling positive whose detection algorithm is accelerating production of CO_2_, the confirmation of presence of a steep rise of the bottle graph may help differentiate true-positive and false-positive samples. In our study, however, most of the identified bacteria were C. acnes, and the graph patterns that could be evaluated were limited to Anaerococcus prevotii, *Bacillus* sp., Staphylococcus (coagulase-negative staphylococci [CNS]), Streptococcus pyogenes, and S. pneumoniae. Thus, it remains possible that different graph patterns can be obtained with other bacterial species. C. acnes is the most commonly recovered organism in bacterial screenings of PCs. It is a ubiquitous skin commensal, considered harmless in the context of usual exposure in healthy individuals (21). However, it has been associated with clinically significant opportunistic infections in the setting of direct exposure through transfusion, surgical procedures, or indwelling devices, particularly in immunosuppressed individuals (22). Therefore, contamination of C. acnes in blood products should be treated carefully.

In the bottles inoculated with S. pneumoniae, 12 h after confirmation of the positive reaction in BacT/Alert 3D, some samples showed bacterial counts below the detection limit in BPN bottles despite the steep rise graph pattern. Furthermore, in the subculture, one sample gave a negative result. We hypothesize it occurred because bacteria lost their viability due to autolysis. Thus, in case a true-positive reaction is suspected due to the bottle graph pattern but the identification test is negative, there is a need to consider the possibility of bacterial autolysis and, therefore, consider the use of an alternative method for bacterial identification, such as 16S rRNA gene sequence analysis to detect nonviable bacterial genes. However, we need to keep in consideration the low sensitivity of the molecular methods (23). Other alternatives, such as Gram staining, a basic and important method to confirm the high concentrations of nonviable bacteria, are available and used in several countries, but we did not apply them because of the need to identify the contaminating bacteria.

There were also some false-positive samples whose detection algorithm was high initial CO_2_ content. The possible reasons of false-positive reaction in BacT/Alert 3D are (i) affection by the temperature change in the lab or the voltage fluctuations/noise, (ii) variations in the sensitivity of the bottle cells dependent on characteristics of the source of light, (iii) variations of the optical reflectance of the individual bottles, (iv) affection by the sample inoculated to the bottle, and (v) delayed loading of the bottle into the BacT/Alert 3D system after sampling. In our cases, the culture bottles were immediately loaded into the system, and it is unlikely that there was an elevation of the initial CO_2_ content. Thus, these cases may have resulted in false-positive reactions due to a combination of factors such as the detection sensitivity of the bottle cell and the reflectance of the bottle, resulting in an incidental increase of the default value of the scatter reflection, giving a positive signal.

From July 2015, when the system was implemented in our lab, to March 2016, we observed an incidence of false-positive reactions as high as 0.32%, so measures were taken to identify the reasons and to implement corrective actions based on the possible reasons described above. First, we improved the lab environment, especially the temperature, preventing direct wind from blowing into the BacT/Alert 3D system. Second, to prevent changes in temperature inside the drawer of the BacT/Alert 3D at the time of bottle loading and removal from the cells, the date of loading into each drawer was fixed. If an incubator module was extensively used for bottle loading or removal, it was not used in the next day. Third, the racks containing aberrant bottle cells were changed. One drawer of the BacT/Alert 3D carries 3 racks, with 20 bottle cells each. If a false-positive reaction was repetitively observed in the same bottle cell, the cell was considered aberrant, and the manufacturer was requested to exchange the rack. By implementing these corrective measures, the false-positive rate decreased from 0.32 to 0.05% or less after April 2016. A similar experience was reported in Australia (15), where the false-positive rate could be reduced by arranging the bottle loading pattern to minimize temperature fluctuations. According to other studies, the rate of false positives in the BacT/Alert system caused by instrument error, using both anaerobic and aerobic bottles, is reported to be 0.02 to 0.28% (13). Comparatively, our rate was similar or even lower. However, this may be dependent on the different conditions of the daily screening bacterial testing from that of the quality control testing. In our quality control sterility testing, the blood products are preserved for longer periods before sampling, which may allow bacterial growth or may affect the CO_2_ kinetics; the manipulation is done in strictly controlled clean rooms and not on a daily basis. These facts may influence the false-positive/-negative reactions in BacT/Alert 3D.

In 2018, 815,823 PC bags were supplied in Japan. Since the false-positive rate in the period from April 2018 to July 2018 was 0.01% in our study, if the screening test by BacT/Alert 3D is implemented for all PC products, around 100 PC bags will be discarded annually due to a false-positive reaction. However, the new bacterial detection system, BacT/Alert Virtuo, with an improved detection algorithm, has been recently released into the market. The introduction of the BacT/Alert Virtuo system will, importantly, contribute to reducing the false-positive/-negative cases, but there is a need to accumulate data to confirm the accuracy of the positive/negative reaction of the system. Although bacterial screening of all PC products has been confirmed to be effective in improving the safety of PC transfusion, many obstacles, dependent on the Japanese societal and medical background, as well as the infrastructure of the blood service, exist and need to be overcome before moving forward.

## MATERIALS AND METHODS

### Quality control sterility testing by BacT/Alert 3D and bacterial identification.

Bacterial testing of blood products by the BacT/Alert 3D for the quality control sterility testing was implemented in Japan in July 2015. We retrospectively analyzed the results of the bacterial testing of RCCs, PCs, and fresh frozen plasma (FFP) at the Japanese Red Cross Kanto-Koshinetsu Block Blood Center, the largest blood center in Japan. Among the blood products which did not pass the infectious disease or biochemical screening tests, 43,374 samples were randomly selected in the period from July 2015 to July 2018. These samples and their coproducts were not supplied to hospitals. The sampling rate is as follows. One sample per 100 bags of RCCs produced was selected, and 1 sample per 500 bags of PCs or FFP produced was selected. From April 2021, the sampling rate of RCCs was changed to 1 sample per 500 bags produced. Then, these samples (38,469 samples of RCCs, 2,041 samples of PCs, and 2,864 samples of FFP) were subjected to quality control sterility testing (Table 1). The storage conditions of the blood products for the quality control sterility testing were as follows. RCCs were stored at a temperature of 2 to 6°C and tested within 54 days after collection. PCs were stored at 20 to 24°C for about 3 days and then refrigerated at 2 to 6°C. They were tested within 19 days after collection. FFP was stored at less than or equal to −20°C and, afterward, thawed and stored at 2 to 6°C. They were tested within 19 days after being thawed. Based on our standard operating procedures (SOPs), 20-mL samples were obtained from the blood bags using a 20-mL syringe (catalog no. JS-S20S1838S; JMS Co., Ltd., Tokyo) after gentle shaking of the bag, and 10 mL was inoculated into an anaerobic bottle (BPN) and the remaining 10 mL into an aerobic bottle (BPA) after disinfection of the inoculation site with 10% povidone-iodine ethanol (Popiyodon Field 10%; Yoshida Pharmaceutical Company Limited, Tokyo, Japan). The culture bottles were loaded into the BacT/Alert 3D system and incubated at 36°C for 10 days.

The BacT/Alert 3D system detects CO_2_ produced through metabolism of the culture medium substrate by bacteria. A red light-emitting LED is irradiated to the CO_2_ sensor on the bottom of the culture bottles every 10 min, and the scatter reflection is read by the detector. According to the strength of the scatter reflection and the change over time, the following parameters can be determined: (i) acceleration of CO_2_ production, (ii) rate of CO_2_ production, and (iii) initial CO_2_ content. If an alteration exceeding the baseline is observed in one of these parameters, the sample signals positive.

When a positive reaction occurred, the culture bottle was removed from the BacT/Alert 3D system, and 1 mL of the culture medium was sampled and inoculated into a new culture bottle. Then, the bottle was incubated for additional days in the BacT/Alert 3D system. If the second bottle signaled positive, then it was removed from BacT/Alert 3D system and sent to an external laboratory for bacterial identification. Even if the second bottle had been negative for 10 days, it was sent to the same laboratory as the first bottle for bacterial identification. In the external laboratory, the positive bottle was subcultured on several kinds of agar plates. In case colonies developed, the identification test was conducted. In case no colonies developed, the sample was defined as negative.

Based on the bacterial identification test, the result of the BacT/Alert 3D was considered a true positive in the case that a bacterium was identified and a false positive in cases where no colonies developed. The following analyses were conducted: (i) confirmation of the detection algorithm of positive reactions in the BacT/Alert 3D, including accelerating production of CO_2_, high rate of CO_2_ production, or high initial CO_2_ content; and (ii) analysis of the bottle graph pattern of positive reactions and the difference between true-positive and false-positive bottles. Some of the negative reactions were also analyzed to compare with the positive reactions.

### Analysis of the bottle graph pattern of the bottles inoculated with Streptococcus pneumoniae.

In addition to the analysis of the results of quality control sterility testing, we conducted the following experiment to evaluate the bottle graph pattern of S. pneumoniae, a bacterium known to autolyze. S. pneumoniae NBRC 102642 was cultured on sheep blood agar medium (Nissui plate 51001; Nissui Pharmaceutical Co., Ltd., Tokyo) at 36°C for 24 h. The colonies were suspended in phosphate-buffered saline (PBS) (pH 7.4) at a target density of 10^8^ CFU/mL by the McFarland turbidity method using DensiChek Plus (bioMérieux). A dilution series was then prepared with the PBS, and the bacterial sample was adjusted to about 4.0 × 10^2^ CFU/mL. The sample was spread on the sheep blood agar medium and cultured at 36°C for 24 h to count the number of bacterial colonies and confirm the bacterial concentration. Three different lots were prepared for each of RCCs, PCs, and FFP. To equalize the volume of all samples, 50 mL of each product was aseptically transferred into small bags (catalog no. BB-T008FJ; Terumo Corporation, Tokyo) using the aseptic bonding device TSCD-II (catalog no. ME-SC203A; Terumo Corporation). The bacterial sample containing about 4.0 × 10^2^ CFU/mL was inoculated into the 9 bags, giving a final dose of bacteria of approximately 4 CFU/mL. Samples (20 mL) were obtained from these bags and inoculated, 10 mL each, into BPN and BPA culture bottles by the same procedure as the quality control sterility testing. Then, the bottles were cultured in the BacT/Alert 3D system.

The positive bottles were removed from the BacT/Alert 3D 12 h after the positive reaction was confirmed, and 0.1 mL of the positive medium was obtained. Then, 10-fold serial dilutions of the medium were prepared using PBS (pH 7.4) and cultured on sheep blood agar medium at 36°C for 24 h for the quantification of bacteria. In case the bacterial count was below the detection limit, the positive medium was analyzed by 16S rRNA gene sequencing to confirm whether S. pneumoniae was identified. From 1 mL of the positive medium, 60 μL of bacterial DNA sample was extracted, and the DNA in 1 μL of the sample was amplified by PCR with TaKaRa Ex Taq (TaKaRa Bio, Inc., Shiga, Japan). Then, the amplified products were sequenced using BigDye Terminator v1.1 cycle sequencing kit (Applied Biosystems, Foster City, CA, USA) and ABI Prism 3130xl genetic analyzer (Applied Biosystems). Identification was performed using comparative analysis with nucleotide BLAST on the National Center for Biotechnology Information server (https://blast.ncbi.nlm.nih.gov). Also, another 2 mL of the positive medium was transferred into a new culture bottle and further cultured in BacT/Alert 3D to confirm whether the second bottle signaled positive.

### Data availability.

The data related to the manuscript are openly available at https://figshare.com/articles/dataset/The_data_related_to_sterility_test_by_BacT_ALERT_3D/17150813/1. These data include total number of the samples and culture-positive cases in the quality control sterility testing and records of preparation of the bacterial sample in Streptococcus pneumoniae inoculation test.
